# Tramadol induced hypoxia signaling and paraptosis-like cell death in breast cancer cells via HIF-1α and ATF4 dependent pathways

**DOI:** 10.1080/13510002.2025.2588866

**Published:** 2026-01-12

**Authors:** Zih-Syuan Wu, Shih-Ming Huang, Yi-Hsuan Huang

**Affiliations:** a Graduate Institute of Life Sciences, College of Biomedical Sciences, National Defense Medical University, Taipei City, 114, Taiwan, Republic of China; b Graduate Institute of Biochemistry, College of Biomedical Sciences, National Defense Medical University, Taipei City, 114, Taiwan, Republic of China; c Department of Anesthesiology, Tri-Service General Hospital, College of Medicine, National Defense Medical University, Taipei City, 114, Taiwan, Republic of China

**Keywords:** Tramadol, ER stress, HIF-1α, reactive oxygen species, paraptosis, ATF4, CHOP, postoperative pain, cytoplasmic vacuolization

## Abstract

**Objectives:**

Tramadol, a clinically approved analgesic widely used for managing postoperative pain, has recently been shown to possess anticancer properties in several tumor models, especially in breast cancer. In this study, we explored the intricate molecular mechanisms by which tramadol induces cytotoxicity in breast cancer cell lines.

**Methods:**

Two invasive ductal carcinoma lines MCF-7 and MDA-MB-231 were used to verify the molecular cytotoxicity of tramadol using cell viability analysis, flow cytometry analysis, real-time polymerase chain reaction, western blotting, Seahorse biogenetic, and transmission electron microscopy analyses.

**Results:**

Our findings demonstrate that tramadol induces the normoxic stabilization and nuclear translocation of hypoxia-inducible factor- 1 alpha (HIF-1α) to activate hypoxia responsive genes. Concurrently, tramadol triggers endoplasmic reticulum (ER) stress and activates the p-eIF2α/ATF4/CHOP signaling axis, leading to the generation of reactive oxygen species, impaired autophagy, mitochondrial dysfunction, including mitochondrial membrane depolarization and the decline of ATP production, cytoplasmic vacuolization, and lipid droplet accumulation which is characteristics of paraptosis-like cell death. Notably, the knockout of HIF-1α or ATF4 significantly reduced tramadol-induced cytotoxicity, highlighting their crucial roles in mediating these cellular responses.

**Conclusion:**

Tramadol induced breast cancer cell death via paraptosis which highlights its therapeutic potential in targeting resistant cancer subtypes such as triple-negative breast cancer.

## GRAPHICAL ABSTRACT

**Figure:**
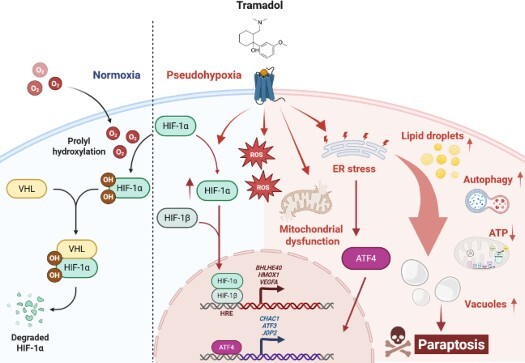

## Introduction

Breast cancer is the most common cancer among women with the five-year survival rate for breast cancer approaching 90% [1]. As patients with breast cancer are living longer, the long-term complications associated with treatment and surgery are increasingly becoming evident [2]. 19.5% to 21.8% of breast cancer survivors experience persistent moderate to severe pain [3]. Compared with their pain-free counterparts, breast cancer survivors dealing with chronic pain report significantly worse scores on assessments of mental health, social functioning, and vitality [4]. Prevalence of post-breast cancer pain has a substantial impact on daily activities and overall quality of life [3]. Addressing these issues is crucial for improving the long-term well-being of breast cancer survivors.

Cancer pain arises not only from the direct effects of tumors but also as a consequence of surgical and pharmacological interventions. Effective pain management remains a critical component of cancer care, as it directly impacts patients’ quality of life [5, 6]. Among analgesics, opioids have received particular attention due to their dual role in pain control and potential effects on tumor behavior. Several preclinical and retrospective clinical studies have reported an association between perioperative opioid administration, perioperative immunosuppression, and cancer progression [7]. This proposed risk is supported by observations that the μ-opioid receptor is overexpressed in several human cancers, including breast cancer, where it may contribute to enhanced tumor growth and metastasis [8]. Nevertheless, the relationship between opioid exposure and cancer progression appears to be complex and context-dependent. Preclinical evidence also indicates that certain opioids can exert antitumor effects. For instance, prolonged morphine treatment has been shown to suppress the proliferation of BT474 human breast cancer cells by modulating the ErbB signaling network [9]. Likewise, opioid ligands acting on distinct receptor subtypes have demonstrated dose-dependent inhibitory effects on the growth of MCF-7 breast cancer cells – an effect reversed by co-administration of the opioid receptor antagonist naloxone [10]. Clinically, emerging evidence suggests that intraoperative opioid administration may even confer protective benefits, such as improved recurrence-free survival in patients with triple-negative breast cancer (TNBC) [11]. Postoperative administration of tramadol has been associated with a lower incidence of cancer recurrence and mortality in patients undergoing breast cancer surgery [12].

Tramadol is a centrally acting analgesic widely accepted for treating moderate postoperative pain. It is commonly used for acute pain management following breast cancer surgery, alongside with a lower risk of tolerance, dependence, and respiratory depression [13, 14]. Tramadol exerts its analgesic effects through dual mechanisms: it binds to μ-opioid receptors and inhibits the reuptake of noradrenaline and serotonin [15]. Both tramadol and its principal metabolite, *O*-desmethyltramadol (M1), exhibit opioid-like effects by binding to μ-opioid receptors in the central nervous system. However, the binding affinity of tramadol for μ-opioid receptors is approximately 6,000 times lower than that of morphine, whereas the affinity of M1 is approximately 20 times lower [16]. Recent studies have demonstrated that tramadol has anticancer effects on breast cancer both *in vitro* and *in vivo* simultaneously [17, 18]. Notably, clinical reports indicate that the postoperative use of tramadol is associated with a reduced risk of breast cancer recurrence [12]. Tramadol combined with doxorubicin demonstrated synergistic anticancer effects in MDA-MB-231 cells, a model of TNBC, as well as specific synergistic outcomes in MCF-7 cells, which are estrogen receptor-positive breast cancer cells [19]. Furthermore, tramadol was found to induce mitochondrial dysfunction in endometrial cancer cells, leading to a decrease in the oxygen consumption rate and an increase in the generation of reactive oxygen species (ROS) [20]. Although these findings are promising, the precise molecular mechanisms underlying the antitumor effects of tramadol in breast cancer remain largely unexplored.

Many studies have shown that while chemotherapy can temporarily induce apoptosis in cancer cells, cancer cells may survive and repopulate due to incomplete or inefficient apoptosis [21, 22]. These findings also indicate that apoptosis does not represent a strict point-of-no-return, as the process can reverse even at advanced stages. Once nuclear fragmentation occurs, apoptosis becomes irreversible [23]. Furthermore, both intrinsic resistance to apoptosis and acquired resistance after treatment (for example, the development of multidrug resistance following breast cancer chemotherapy) present significant challenges in effective cancer therapy. Therefore, identifying alternative cell death mechanisms could be a key to overcoming apoptosis resistance. There are two main types of alternative cell death pathways: caspase-dependent and caspase-independent, including anoikis, autosis, ferroptosis, pyroptosis, necroptosis, and paraptosis [24]. Recent studies have shown that several natural compounds can trigger paraptosis in different tumor cell lines, including breast cancer cell lines [25, 26]. Paraptosis was recently described as a form of programmed cell death (PCD) characterized by features distinct from those of apoptotic cell death. The hallmark of paraptosis is extensive cellular vacuolization, which is generated from either endoplasmic reticulum (ER) stress or mitochondrial swelling [24, 26]. It is well established that increased ROS production contributes to mitochondrial dysfunction and/or ER stress, which is a prerequisite for the formation of paraptotic vacuoles.

ER stress is known to accumulate as part of the unfolded protein response (UPR) and is a primary driver of ROS production and autophagy [27, 28]. Three primary UPR pathways are involved: inositol-requiring enzyme-1α (IRE1), activating transcription factor 6 (ATF6), and protein kinase RNA-like endoplasmic reticulum kinase (PERK). Each pathway plays a distinct role in managing cellular stress. Activated ATF6 functions as a transcription factor, promoting the expression of ER chaperones and XBP1 [29, 30]. When activated, IRE1 splices XBP1 mRNA to produce the spliced XBP1 protein (XBP1s), which translocates to the nucleus to regulate the transcription of ER-resident chaperones and genes involved in lipogenesis and ER-associated degradation [31]. Moreover, PERK activation leads to the phosphorylation of eukaryotic initiation factor 2α (p-eIF2α), which inhibits general protein synthesis while selectively allowing the translation of activating transcription factor 4 (ATF4) [27, 32]. ATF4 then moves to the nucleus, where it drives the transcription of various genes essential for ER quality control. The ER and mitochondria are closely connected organelles through mitochondria-associated membranes. Numerous studies have confirmed that ER stress triggers mitochondrial dysfunction [33–35].

Hypoxia-inducible factors (HIFs) are transcription factors that serve as key regulators of oxygen homeostasis, orchestrating various adaptive responses to hypoxic conditions [36, 37]. The HIF-α subunit acts as the primary hypoxia sensor. HIFs function as heterodimers, comprising a hypoxia-sensitive HIF-α subunit and a constitutively expressed HIF-β subunit. The stability of HIF-α proteins is regulated post-translationally by prolyl hydroxylase domain (PHD)-containing enzymes [38]. Under normoxic conditions, HIF-1α and HIF-2α are continuously hydroxylated by PHDs at specific proline residues, marking them for rapid degradation via the von Hippel-Lindau (pVHL)-ubiquitin-proteasome pathway [39]. In mild hypoxia, PHD activity is inhibited, allowing HIF-α proteins to escape pVHL-mediated degradation, stabilize, translocate to the nucleus, and dimerize with HIF-β. This forms the HIF transcriptional complex, which binds to hypoxia response elements (HREs) in target gene promoters. HIF-regulated target genes play crucial roles in cellular and systemic adaptations to hypoxia, including metabolic reprogramming, resistance to apoptosis, angiogenesis, invasion, and metastasis [40]. Overexpression of HIF-1α has been linked to poor prognosis in several cancers, including breast, cervical, oropharyngeal, ovarian, and endometrial cancers [40]. However, recent studies have reported an association between HIF-1α overexpression and reduced mortality in patients with head and neck squamous cell carcinoma and non-small-cell lung cancer [41]. This apparent discrepancy may stem from the limitations of current clinical methods used to assess protein levels in cancer cells. Immunohistochemical analysis, the most common technique for measuring protein expression, does not provide information on potential mutations or post-translational modifications that could influence HIF-1α functions. As a result, variations in HIF-1α activity rather than expression alone may contribute to its differing prognostic significance across cancer types.

Hence, in this study, we aimed to clarify which type of cell death is induced by tramadol in breast cancer cells, to investigate whether this effect may involve a nonapoptotic form of cell death such as paraptosis, and to determine the underlying molecular mechanisms, particularly the involvement of HIF-1α and ATF4 dependent signaling. Understanding these mechanisms could provide valuable insights into the potential of tramadol as a therapeutic agent in breast cancer and its ability to increase the efficacy of existing treatments.

## Methods and materials

### Cell culture and reagents

MDA-MB-231 (HTB-26™) cells were purchased from American Type Culture Collection (Manassas, VA, USA). MCF-7 (BCRC-60436) cells were purchased from the Bioresource Collection and Research Center (Hsinchu, Taiwan). MDA-MB-231 cells were cultured in Dulbecco's modified Eagle's medium (DMEM) (catalog no.: 10–013-CM; Corning, New York, NY, USA) supplemented with 10% (v/v) fetal bovine serum (FBS) (catalog no.: A52567-01; Gibco Life Science, Paisley, Scotland) and 1% penicillin – streptomycin (catalog no.: 15140-122; Gibco Life Science). MCF-10A cells were cultured in DMEM/F12 (catalog no.: 10–092-CV; Corning) supplemented with 10% FBS and 1% penicillin – streptomycin. MCF-7 cells were cultured in minimum essential medium (MEM) (catalog no.: 61100–061; Gibco Life Science) supplemented with 2 mM L-glutamine and Earle's balanced salts, which contained 1.5 g/L sodium bicarbonate, 0.1 mM nonessential amino acids, 1.0 mM sodium pyruvate, 10% (v/v) FBS and 1% (w/v) penicillin – streptomycin.

Tramadol (catalog no.: 1672600) was obtained from United States Pharmacopeia (Rockville, Maryland, USA). 2′,7-dichlorofluorescein diacetate (catalog no.: 35845), NAC (catalog no.: A0737), and CHX (catalog no.: C4859) were obtained from Sigma Aldrich (St. Louis, MO, USA). GSK260414 (catalog no.: HY-18072), ferrostatin-1 (catalog no.: HY-100579), necrostatin-1 (catalog no.: HY-15760), and Z-VAD-FMK (catalog no.: HY-16658B) were obtained from MedChemExpress (Monmouth Junction, NJ, USA). MitoSox Red (catalog no.: M36008) was obtained from Thermo Fisher Scientific (Waltham, MA, USA).

### Cell viability and cytotoxicity analysis

Cells (5 × 10³ per well) were seeded into 96-well plates and incubated overnight at 37°C with 5% (v/v) CO_2_. The following day, the cells were treated with various concentrations of tramadol. Cytotoxicity was assessed using the CellTox™ Green Cytotoxicity Assay (catalog no.: G8743; Promega, Madison, WI, USA), whereas cell viability was measured using the CellTiter-Glo® 2.0 Assay (catalog no.: G9241; Promega). Fluorescence or luminescence signals were detected using a Varioskan LUX plate reader (Thermo Fisher Scientific).

### RNA isolation, RNA-Seq, and RT–qPCR

For RNA isolation, cells were washed twice with PBS and lysed with TRIzol reagent (catalog no.: 15596026; Sigma Aldrich) according to the manufacturer's protocol, and then, 1 μg of total RNA was reverse transcribed using MMLV reverse transcriptase (catalog no.: PT-MMLV; Protech Technology, Taipei, Taiwan) at 42°C for 60 min. The qPCR primers are listed in Table 1.

**Table 1.** Primer sequences were used for qPCR analysis in this study

Target Gene (Accession Code)^a^	Forward Primer (5'−3’)	Reverse Primer (5'−3’)
*HIF-1α* (NM_181054.3)	GAACGTCGAAAAGAAAAGTCTCG	CCTTATCAAGATGCGAACTCACA
*BHLHE40* (XM_054348161.1)	GACGGGGAATAAAGCGGAGC	CCGGTCACGTCTCTTTTTCTC
*HMOX1* (NM_002133.3)	AAGACTGCGTTCCTGCTCAAC	AAAGCCCTACAGCAACTGTCG
*VEGFA* (NM_001025370.3)	AGGGCAGAATCATCACGAAGT	AGGGTCTCGATTGGATGGCA
*ACTB* (NM_001101.5)	ACAGGAAGTCCCTTGCCATC	CAGTGTACAGGTAAGCCCTGG
*ATF4* (NM_001675.4)	TTCTCCAGCGACAAGGCTAAGG	CTCCAACATCCAATCTGTCCCG
*CHAC1* (XM_054378768.1)	CCTGAAGTACCTGAATGTGCGAGA	GCAGCAAGTATTCAAGGTTGTGGC
*DDIT3* (NM_001413642.1)	GGAAACAGAGTGGTCATTCCC	CTGCTTGAGCCGTTCATTCTC
*ATF3* (NM_001206486.2)	CCTCTGCGCTGGAATCAGTC	TTCTTTCTCGTCGCCTCTTTTT
*JDP2* (XM_047430942.1)	CCCAGCCCGTGAAAAGTGA	CGGTGTCGGTTCAGCATCA
*CYP1A1* (NM_001319216.2)	TCGGCCACGGAGTTTCTTC	GGTCAGCATGTGCCCAATCA
*PDCD6IP* (NM_013374.6)	ATGGCGACATTCATCTCGGTG	CGCTTGGGTAAGTCTGCTGG

^a^ NCBI accession numbers were listed below each target gene.

For RNA-seq, 1 μg of total RNA was used for library preparation. Poly(A) mRNA isolation was performed using Oligo(dT) beads. mRNA fragmentation was performed using divalent cations and high temperature. Priming was performed using random primers. First-strand cDNA and second-strand cDNA were synthesized. The purified double-stranded cDNA was then treated to repair both ends, and dA-tailing was added in one reaction, followed by T-A ligation to add adaptors to both ends. Size selection of adaptor-ligated DNA was then performed using DNA Clean Beads. Each sample was then amplified via PCR using the P5 and P7 primers, and the PCR products were validated. Libraries with different indices were then multiplexed and loaded on an Illumina HiSeq/Illumina NovaSeq/MI 2000 instrument for sequencing using a 2 × 150 paired-end configuration according to the manufacturer’s instructions.

### Differential expression analysis and enrichment analysis

Transcript sequences were extracted in FASTA format based on the reference GFF annotation and subsequently indexed for downstream analysis. Quantification of gene and transcript isoform expression was performed using HTSeq (v0.6.1) on the high-quality paired-end reads. Differential gene expression was assessed using the DESeq2 package from Bioconductor (v1.6.3), which estimates dispersion and log₂ fold changes through empirical Bayesian shrinkage. Genes meeting the threshold of adjusted *p*-value (Padj) ≤ 0.05 were deemed statistically significant. To validate these findings, an additional analysis was conducted with EdgeR (v3.4.6), using criteria of |log₂ fold change| > 1 (equivalent to a fold change greater than 2) and Padj < 0.05. To explore associated biological pathways, Gene Set Enrichment Analysis (GSEA) was applied using the hallmark gene set collection ‘h.all.v2024.1.Hs.symbols.gmt’ to identify enriched molecular signatures.

### Hypoxia analysis

Cells were treated with tramadol, followed by staining with Hypoxia Red dye (catalog no.: ENZ-51042; Enzo Life Sciences, Farmingdale, NY, USA) for 30 min at 37 °C, according to the manufacturer's instructions. The hypoxia level of cells was then analyzed by FACSCalibur flow cytometer and Cell Quest Pro software.

### Western blot

After treatment with tramadol, the cells were lysed with radioimmunoprecipitation assay (RIPA) buffer to extract proteins. The protein concentration was measured with a DC protein assay, and 30 µg of protein was loaded onto an SDS–PAGE gel. Following electrophoresis, the proteins were transferred onto PVDF membranes, which were then blocked with 5% (w/v) nonfat milk in TBST for 1 h. The membranes were incubated with primary antibodies overnight at 4°C with gentle shaking, followed by incubation with secondary antibodies for 1 h at room temperature. Primary antibodies against β-actin (catalog no.: sc-47778), GAPDH (catalog no.: sc-47724), p62 (catalog no.: sc-28359), ALG2 (catalog no.: sc-376950), Alix (catalog no.: sc-53540), and α-actinin (ACTN, catalog no.: sc-17829) were obtained from Santa Cruz Biotechnology (Santa Cruz, CA, USA), and primary antibodies against HIF-1α (catalog no.: #14179), PERK (catalog no.: #3192), p-eIF2α (catalog no.: #9721), ATF4 (catalog no.: #11815), CHOP (catalog no.: #2895), ATF6 (catalog no.: #65880), XBP1 (catalog no.: #40435), IRE1 (catalog no.: #3294), H3 (catalog no.: #9715), LC3B (catalog no.: #2775), PARP (catalog no.: #9546), p-ERK (catalog no.: #4370), ERK (catalog no.: #4695), p-p38 (catalog no.: #9211), p-38 (catalog no.: #9212), p-JNK (catalog no.: #4671), and JNK (catalog no.: #67096) were obtained from Cell Signaling Technology (Danvers, MA, USA).

### Immunofluorescence microscopy

Cells were seeded on coverslips, treated as indicated, and fixed in 3.7% (v/v) paraformaldehyde for 15 min. After washing the cells with PBS, permeabilization was carried out with 0.2% (v/v) Triton X-100, followed by blocking with 5% (w/v) BSA for 30 min. The fixed samples were then incubated with primary antibodies overnight, followed by secondary antibodies for 1 h. Cells were next observed using a THUNDER Imager microscope equipped with a 100× objective (Leica, Wetzlar, Germany).

### Subcellular cytoplasmic and nuclear extract preparation

Cells were cultured in 100-mm dishes and treated with tramadol. Cytoplasmic and nuclear extracts were prepared using a Subcellular Protein Fractionation Kit for Cultured Cells (catalog no.:78840; Thermo Fisher Scientific) following the manufacturer's protocol. For the cytoplasmic fraction, the cells were lysed in cytoplasmic extraction buffer at 4°C, and the cytoplasmic supernatant was collected by centrifugation at 14,000 × g for 15 min at 4°C. To obtain the nuclear fraction, the cell pellets were lysed in Nuclear Extraction Buffer at 4°C, and the nuclear supernatant was similarly collected by centrifugation at 14,000 × g for 15 min at 4°C.

### Reactive oxygen species (ROS) assay

We measured intracellular and mitochondrial ROS levels via DCFH-DA and MitoSOX Red staining, respectively. Following treatment with tramadol, the cells were washed twice with PBS and then incubated with 10 μM DCFH-DA or 5 μM MitoSOX Red at 37°C for 30 min in the dark. Then, the cells were washed with PBS and analyzed using a FACSCalibur flow cytometer and Cell Quest Pro software.

### Transmission electron microscopy (TEM) analysis of cellular ultrastructural morphological changes

Cells were seeded in 60-mm culture dishes and treated with tramadol (1 mg/ml). After treatment, the cells were digested and fixed with 2.5% (v/v) glutaraldehyde and 2% (v/v) paraformaldehyde. The cell samples were then stained with 2% uranyl acetate, dehydrated using a graded ethanol series, and treated with propylene oxide as a transitional solvent before being embedded in EPON 812. Ultrathin sections (80 nm thick) were prepared using an ultramicrotome (Ultracut UCT, Leica). Images were captured with a transmission electron microscope (HT7700; Hitachi, Ibaraki, Japan).

### BODIPY staining

Cells were stained with BODIPY 493/503 (5 µg/ml in PBS, protected from light) (catalog no.: D3922; Invitrogen, Carlsbad, CA, USA) for 30 min. Following three washes with PBS, fluorescence intensity was captured using a fluorescence microscope and subsequently analyzed with a FACSCalibur flow cytometer and Cell Quest Pro software.

### Acridine orange (ΑΟ) and DALGreen staining

After the cells were treated with tramadol, AO (catalog no.: A8097; Sigma Aldrich) was applied at a concentration of 1 µg/ml for 30 min. Then, the cells were washed with PBS. The stained cells were subsequently visualized and photographed using a fluorescence microscope.

Autophagy flux was assessed using DALGreen (catalog no.: D675; Dojindo Molecular Technologies, Kumamoto, Japan). Following treatment with tramadol, the cells were washed twice with PBS and incubated with DALGreen in culture medium for 1 h at 37°C. After three additional PBS washes, the fluorescence intensity was analyzed with a FACSCalibur flow cytometer and Cell Quest Pro software.

### Assessments of mitochondrial morphology, mitochondrial membrane potential, and oxygen consumption rate (OCR)

To assess mitochondrial morphology, the cells were treated with tramadol and then stained with 25 nM MitoTracker Red (catalog no.: M7512; Invitrogen) for 15 min at 37°C. The stained cells were subsequently visualized and photographed using a fluorescence microscope.

Mitochondrial membrane potential was assessed using a MitoScreen (JC-1) kit (catalog no.: 551302; BD Pharmingen, San Jose, CA, USA). Following treatment with tramadol, both live and dead cells were collected, and JC-1 solution was added to the cells, followed by a 15 min incubation. The cells were then washed twice with binding buffer, and each sample was analyzed using a FACSCalibur flow cytometer and Cell Quest Pro software.

The OCR was measured using a Seahorse XF24 Analyzer following the manufacturer's protocol (catalog no.: 103015-100; Agilent, Santa Clara, CA, USA). Briefly, after treatment with tramadol, the culture medium was replaced with sodium bicarbonate-free DMEM (pH 7.4), and the cells were cultured for 1 h. The OCR was sequentially assessed before and after the addition of oligomycin (1 μM), FCCP (0.5 μM), and rotenone/antimycin A (0.5 μM).

### Lentivirus infection

Guide RNAs targeting HIF-1α (HSPD0000018711 or HSPD0000018713), ATF4 (HSPD0000003012), and PDCD6IP (HSPD0000059446) were purchased from Sigma Aldrich (in the LV01 all-in-one lentiviral plasmid). A lentivirus expressing specific constructs was produced by transfecting HEK293 cells in 6-well plates at a 1:1:0.1 ratio of lentiviral vector:pMD2.G:psPAX2 using TransIT-LT1 transfection reagent (Mirus, Madison, WI, USA). The lentivirus was subsequently filtered through a 0.45 μm PES filter, and 1 ml of media containing the lentivirus was subsequently added to 6-well plates containing cells along with polybrene (10 μg/ml). Puromycin was used to select infected cells.

### Statistics

Bar graphs include individual data points to represent biological replicate numbers. The figure legends for the line graphs specify the number of biological replicates. The data are expressed as the mean ± standard error of the mean (SEM). Statistical significance was determined using GraphPad software with one-way ANOVA and Tukey’s multiple comparisons, two-way ANOVA with Dunnett’s multiple comparisons, or a two-tailed Student’s t test, as appropriate. The error bars on the graphs represent the SEM, and the significance between samples is denoted as *p < 0.05, **p < 0.01, ***p < 0.001, and ****p < 0.0001.

## Results

### Tramadol selectively reduced cancer cell viability and induced transcriptional reprogramming

To assess the cytotoxic potential of tramadol, we first evaluated cell viability in MCF-7, MDA-MB-231, and non-tumorigenic MCF-10A breast epithelial cells using MTT assays. A dose-dependent suppression of metabolic activity was observed in both cancer cell lines, with MCF-7 cells exhibiting approximately 1.5-fold greater susceptibility than MDA-MB-231 cells. In contrast, MCF-10A cells exhibited relative resistance to tramadol, indicating a potential therapeutic window for cancer-selective cytotoxicity (Figure 1A). To explore the molecular basis of tramadol's cytotoxicity, RNA-seq analysis was performed on tramadol-treated MCF-7 and MDA-MB-231 cells. Differential expression analysis revealed extensive transcriptomic remodeling: 319 genes were significantly upregulated and 95 were downregulated in MDA-MB-231 cells, while MCF-7 cells displayed 210 upregulated and 195 downregulated genes (Figure 1B). Gene set enrichment analysis (GSEA) further identified consistent enrichment of pathways related to hypoxia response, the unfolded protein response (UPR), and reactive oxygen species (ROS) signaling in both cell types (Figure 1C).

**← Figure 1.**
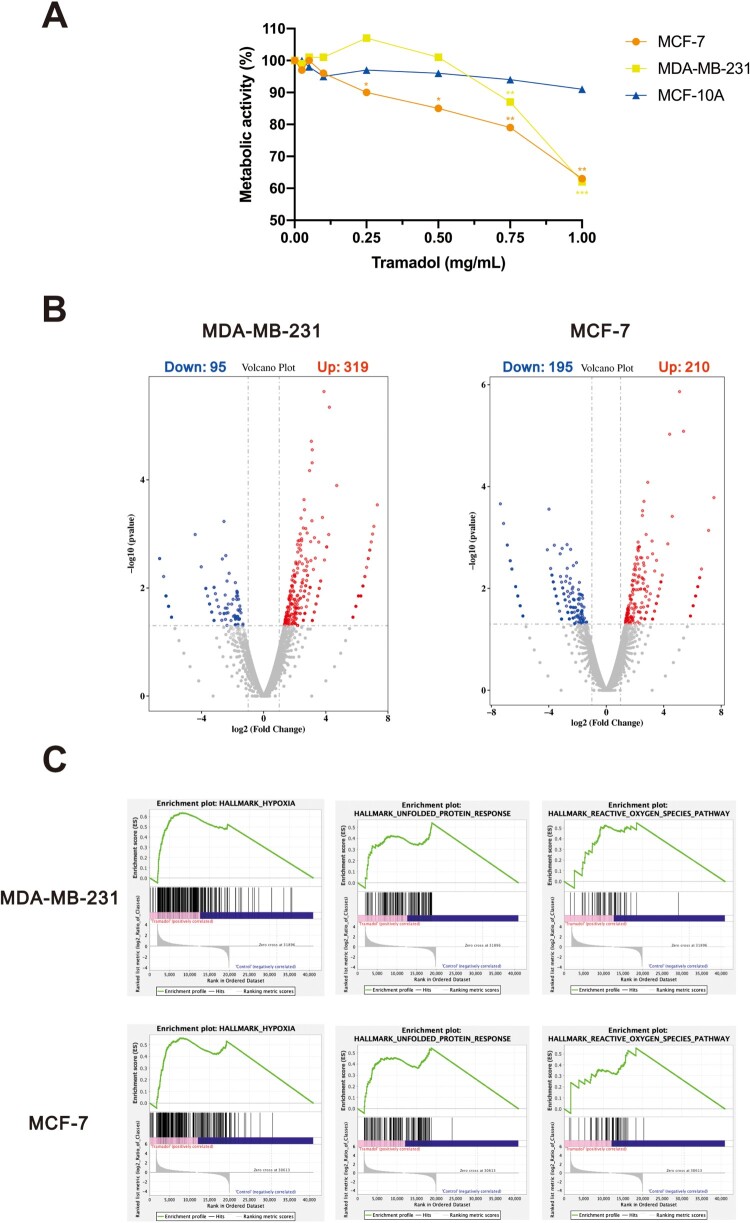
Tramadol impaired cell viability and induced transcriptional and pathway-level alterations in breast cancer cells. A Cell viability was measured using the MTT assay after treating MDA-MB-231, MCF-7, and MCF-10A cells with escalating doses of tramadol for 24 h. Results represent the mean ± SEM from three biologically independent replicates. Statistical comparisons were conducted using two-way ANOVA followed by Dunnett’s post hoc test (*p < 0.05, **p < 0.01, ***p < 0.001 vs. control). B Volcano plots illustrating changes in gene expression profiles in MDA-MB-231 and MCF-7 cells after 24-hour exposure to 0.5 mg/mL tramadol. Differentially expressed genes were classified as upregulated (red) or downregulated (blue) based on a threshold of |log₂ fold change| > 1 and adjusted *p*-value < 0.05. C Hallmark pathway analysis was conducted using GSEA to compare tramadol-treated and untreated cells. Key enriched signatures included hypoxia signaling, ER stress/unfolded protein response, and oxidative stress pathways.

### Tramadol triggered hypoxia-like responses and stabilized HIF-1α under normoxic conditions in breast cancer cells

To verify whether tramadol could elicit hypoxia-like cellular responses, we utilized a hypoxia-sensitive probe that emits fluorescence in the presence of nitroreductase activity, a biochemical hallmark of hypoxic environments. Flow cytometric analysis showed a concentration-dependent elevation in hypoxia-associated fluorescence following tramadol exposure in both MDA-MB-231 and MCF-7 cells, implying that tramadol induced a pseudo-hypoxic state despite ambient oxygen levels (Figure 2A). Our prior work had demonstrated that tramadol increases HIF-1α protein expression in normoxic breast cancer cells [19]. To determine whether this upregulation resulted from enhanced protein stability, we inhibited translation using cycloheximide (CHX) and monitored the degradation kinetics of HIF-1α protein. In both breast cancer cell lines, tramadol treatment significantly extended HIF-1α half-life, supporting the conclusion that it impedes normoxic degradation of the protein (Figure 2B).

**← Figure 2.**
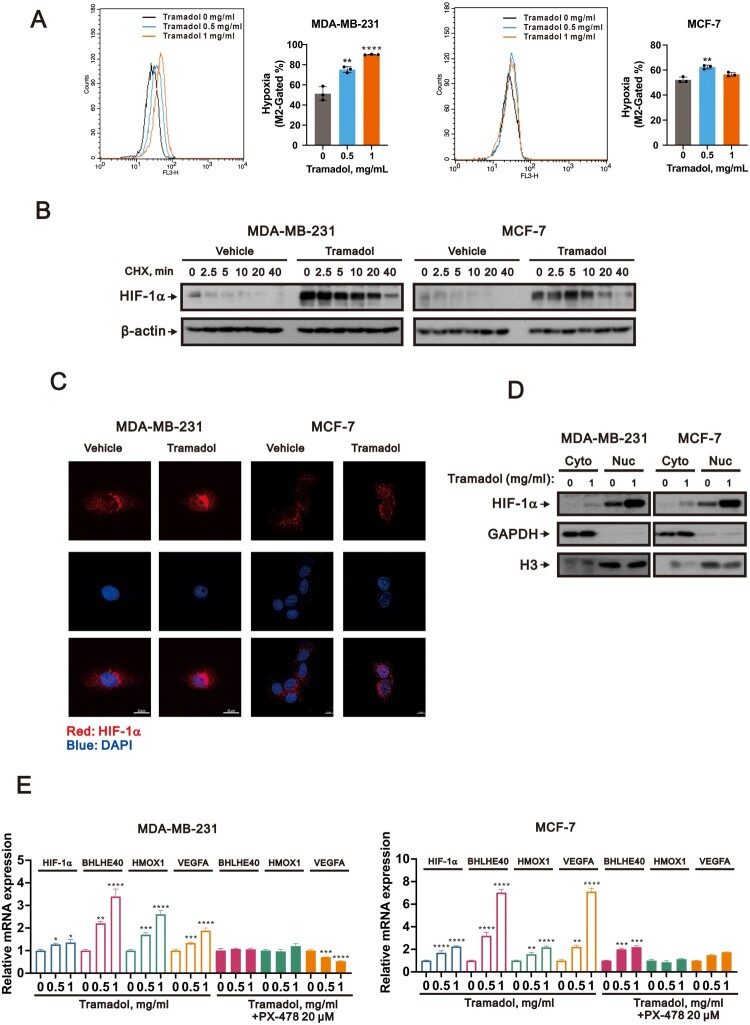
Tramadol promoted hypoxia signaling in a dose-dependent manner by stabilizing HIF-1α, enhancing its nuclear translocation, and upregulating HIF-1α target gene expression in breast cancer cells. A Hypoxia levels were elevated in MDA-MB-231 and MCF-7 cells following short-term tramadol exposure. Cells were incubated with 0, 0.5, or 1 mg/mL tramadol for 4 h, stained with a hypoxia-sensitive fluorescent dye (Hypoxia Red), and analyzed via flow cytometry. Quantification of the hypoxic population (M2 gate) is shown as mean ± SEM from three independent experiments. Statistical significance was assessed using one-way ANOVA with Tukey’s multiple comparisons (**p < 0.01, ****p < 0.0001 vs. untreated control). B Tramadol prolonged the stability of HIF-1α protein. MDA-MB-231 and MCF-7 cells were treated with 1 mg/mL tramadol or vehicle for 4 h prior to cycloheximide (CHX) addition. Protein degradation kinetics of HIF-1α were monitored at various time points (0–40 min) by western blot. β-actin served as the internal loading control. C Immunofluorescence staining revealed tramadol-induced nuclear localization of HIF-1α in breast cancer cells. MDA-MB-231 and MCF-7 cells were exposed to 1 mg/mL tramadol or vehicle control for 4 h, followed by staining with an anti-HIF-1α antibody (red) and nuclear counterstaining with DAPI (blue). Representative fluorescence images were acquired using a fluorescence microscope. Scale bar: 10 μm. D Subcellular fractionation followed by western blot analysis further confirmed the nuclear enrichment of HIF-1α following tramadol treatment (1 mg/mL, 4 h). Nuclear (Nuc) and cytoplasmic (Cyto) protein fractions were isolated from MDA-MB-231 and MCF-7 cells. GAPDH and histone H3 served as markers for cytoplasmic and nuclear compartments, respectively. E Tramadol induced the transcription of HIF-1α and its downstream targets BHLHE40, HMOX1, and VEGFA in a dose-dependent manner. Cells were treated with 0, 0.5, or 1 mg/mL tramadol for 4 h, with or without the HIF-1α inhibitor PX-478 (20 μM). Gene expression was measured by qPCR, normalized to ACTB, and shown as mean ± SEM (n = 3). Statistical analysis was performed using one-way ANOVA with Tukey’s multiple comparisons (*p < 0.05, **p < 0.01, ***p < 0.001, ****p < 0.0001 vs. untreated control).

As HIF-1α exerts its function by regulating gene transcription within the nucleus, we examined its intracellular distribution following tramadol treatment. Immunofluorescence staining revealed strong nuclear localization of HIF-1α in tramadol-treated breast cancer cells, marked by intensified nuclear red signals in both MDA-MB-231 and MCF-7 cells (Figure 2C). These observations were further validated by nuclear and cytoplasmic fractionation followed by western blotting, which confirmed increased nuclear accumulation of HIF-1α protein upon tramadol exposure (Figure 2D). These results suggest that tramadol not only stabilized HIF-1α under normoxia but also promoted its translocation into the nucleus, an essential step for its transcriptional activity. To assess whether nuclear HIF-1α retained its transcriptional function, we performed quantitative polymerase chain reaction (qPCR) analysis to evaluate expression levels of known downstream targets, including *BHLHE40*, *HMOX1*, and *VEGFA*. Tramadol treatment led to a dose-dependent increase in the expression of all three genes in both MDA-MB-231 and MCF-7 cells (Figure 2E). To determine the dependency on HIF-1α activity, we employed PX-478, a pharmacological inhibitor that blocks HIF-1α-mediated transcription. Pre-treatment with PX-478 significantly reduced tramadol-induced upregulation of these genes, indicating that the gene expression changes were indeed mediated through HIF-1α. These data showed that tramadol under normoxia activated HIF-1α signaling by promoting its nuclear localization and target gene expression.

### Tramadol activated the ER stress-associated p-eIF2α/ATF4/CHOP signaling pathway and elevated intracellular ROS levels in breast cancer cells

**Figure 3.**
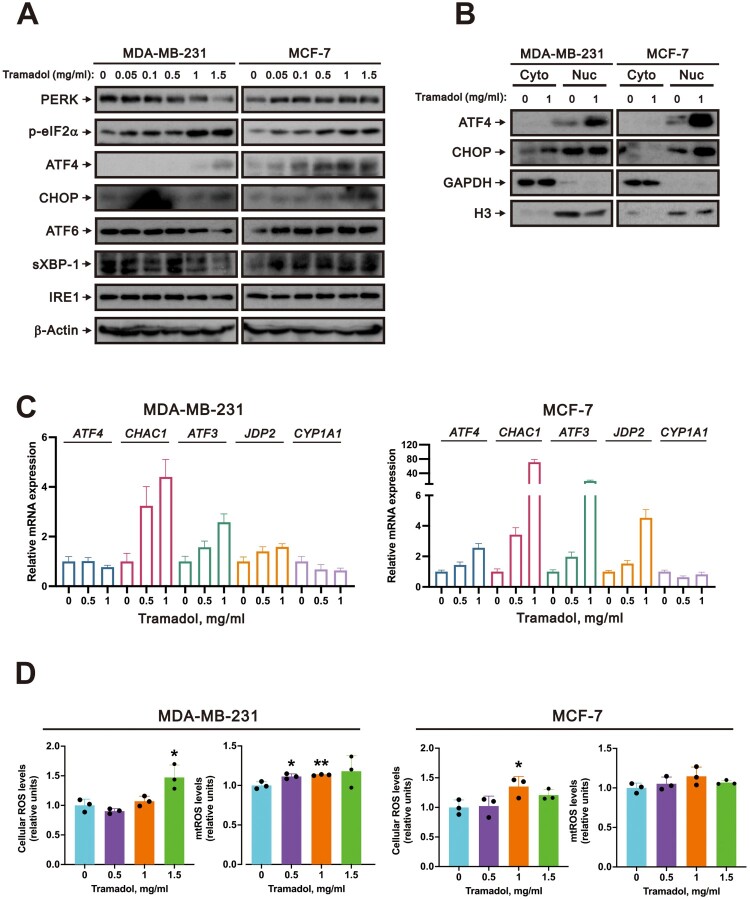
Effects of tramadol on the ER stress pathways and ROS levels in MDA-MB-231 and MCF-7 cells. A MDA-MB-231 and MCF-7 cells were treated with 0, 0.05, 0.1, 0.5, 1, or 1.5 mg/ml tramadol. β-Actin was used as a loading control. B MDA-MB-231 and MCF-7 cells were treated with 0 or 1 mg/ml tramadol. Western blot analysis was performed on the nuclear and cytosolic fractions, with Histone 3 and GAPDH serving as internal controls for the nuclear and cytosolic proteins, respectively. C MDA-MB-231 and MCF-7 cells were treated with 0 or 1 mg/ml tramadol. qPCR analysis was performed to assess the mRNA expression of downstream genes regulated by ATF4. Statistical analysis was performed using one-way ANOVA with Tukey’s multiple comparisons (*p < 0.05, **p < 0.01, ***p < 0.001, ****p < 0.0001 vs. control). D MDA-MB-231 and MCF-7 cells were treated with 0, 0.5, 1, or 1.5 mg/ml tramadol. DCFH-DA staining was conducted to assess intracellular ROS levels, whereas MitoSox Red staining was used to evaluate mitochondrial ROS levels, both of which were analyzed via flow cytometry. Statistical analysis was performed using one-way ANOVA with Tukey’s multiple comparisons (*p < 0.05, **p < 0.01 vs. control).

To investigate the specific UPR pathways activated by tramadol-induced ER stress in breast cancer cells, we performed western blotting to assess the involvement of the three primary UPR pathways. Our western blot results demonstrated that tramadol induced the expression of the IRE1, ATF6, and PERK proteins in MCF-7 cells but downregulated the expression of those proteins in MDA-MB-231 cells. However, the expression of the components of the p-eIF2α/ATF4/CHOP signaling pathway, which is activated by the UPR, was detected in both cell types (Figure 3A). ATF4 also induced the expression of the C/EBP homologous protein (CHOP) transcription factor (Figure 3A). Next, we confirmed through nuclear–cytoplasmic fractionation that tramadol-induced ATF4 translocated to the nucleus (Figure 3B) in both breast cancer cell lines. This translocation is crucial, as it enables ATF4 to function as a transcription factor and regulate the expression of genes involved in the cellular response to ER stress. We then performed qPCR to validate the expression of ATF4 downstream target genes identified in our RNA-seq analysis. The expression of the genes encoding *glutathione-specific gamma-glutamylcyclotransferase 1* (*CHAC1*), *ATF3*, and *Jun dimerization protein 2* (*JDP2*), which was upregulated following treatment with tramadol, as shown in Figure 1c, exhibited a consistent increasing trend (Figure 3C). These findings suggested that tramadol triggered an ATF4-mediated transcriptional response, activating genes crucial for adapting to ER stress.

Since ATF4 and CHOP cooperatively stimulate the expression of genes involved in protein synthesis, the reactivation of translation can lead to ROS overproduction, potentially triggering cell death [42]. Studies have shown that the forced expression of ATF4 and CHOP increases protein synthesis, leading to ATP depletion and oxidative stress, ultimately resulting in cell death [27, 43]. Furthermore, our previous GSEA revealed that treatment with tramadol significantly activated the ROS pathway in the MDA-MB-231 and MCF-7 breast cancer cell lines (Figure 3D). In subsequent experiments, we assessed whether tramadol affects ROS levels in breast cancer cells. The results revealed that tramadol significantly increased cytosolic ROS levels in both MDA-MB-231 and MCF-7 cells, with a notable increase in mitochondrial ROS levels observed only in MDA-MB-231 cells (Figure 3D). This cell-specific mitochondrial ROS response suggested that, compared with MCF-7 cells, MDA-MB-231 cells may be more susceptible to mitochondrial oxidative stress under tramadol treatment.

### Tramadol induced cellular vacuolation and lipid droplet formation in MDA-MB-231 and MCF-7 cells

**Figure 4.**
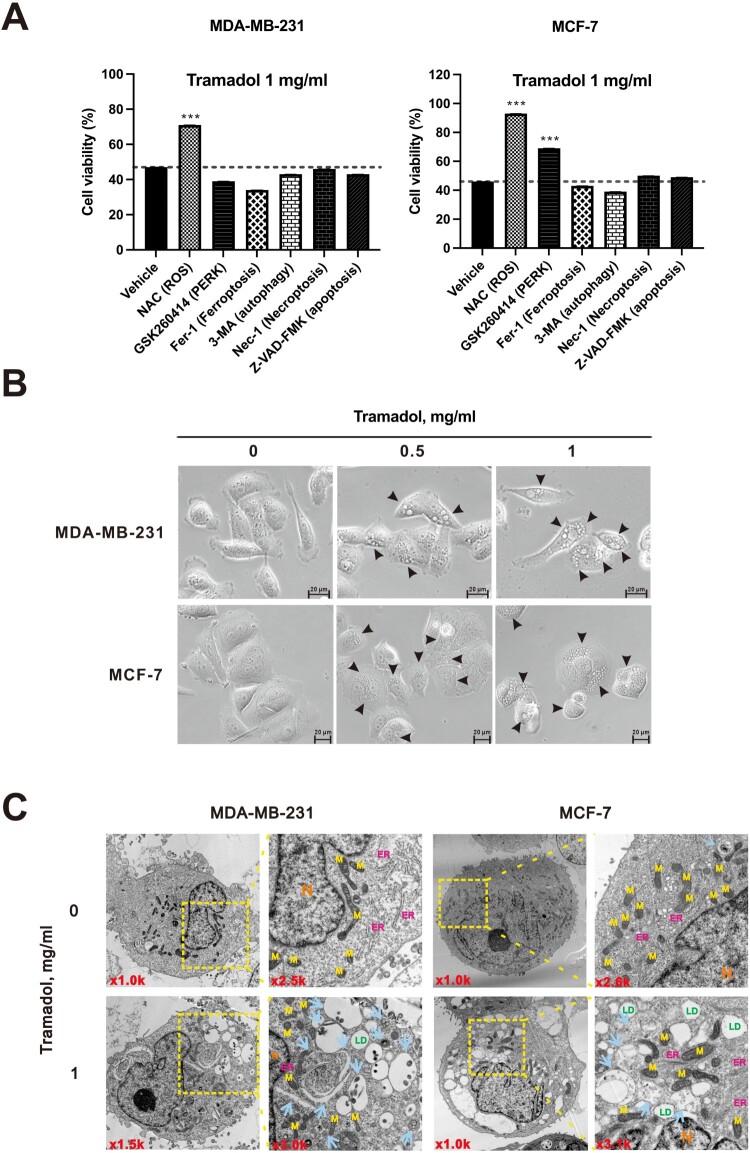
The mechanisms and types of cell death induced by tramadol in MDA-MB-231 and MCF-7 cells. A After treatment with various inhibitors, cells were treated with 1 mg/ml tramadol, and then, cell viability was assessed. Data are shown as mean ± SEM; ***p < 0.001 vs. tramadol alone (two-tailed unpaired Student’s t-test). B Phase-contrast images of breast cancer cells following treatment with 0, 0.5, or 1 mg/ml tramadol. Arrows indicate cytoplasmic vacuoles. C MDA-MB-231 and MCF-7 cells were treated with 1 mg/mL tramadol and then analyzed via electron microscopy. Representative regions (yellow squares) are enlarged and labeled as follows: M, mitochondria; N, nucleus; LD, lipid droplet; ER, endoplasmic reticulum; Arrow, autophagic vacuoles.

Next, we investigated whether tramadol-induced breast cancer cell death could be rescued by various inhibitors of ROS (N-acetylcysteine, NAC), PERK (GSK260414), ferroptosis (Fer-1), autophagy (3-MA), necroptosis (Nec-1), and apoptosis (Z-VAD-FMK) and to clarify the type of cell death involved. The results indicated that in MDA-MB-231 cells, tramadol-induced cell death could be rescued by the ROS scavenger NAC (Figure 4A). In contrast, in MCF-7 cells, cell death was rescued by NAC and GSK260414. Notably, none of the inhibitors targeting ferroptosis, autophagy, necroptosis, or apoptosis prevented tramadol-induced cell death in either cell line.

Vacuolation, a defining morphological feature, aligns with paraptosis, which is characterized by cytoplasmic vacuolization from the ER and mitochondrial swelling [44]. Interestingly, phase-contrast microscopy revealed notable morphological changes in breast cancer cells following treatment with tramadol. In both MDA-MB-231 and MCF-7 cells, numerous cytoplasmic vacuoles (black arrows indicate cytoplasmic vacuoles) were observed, which increased in number with higher doses and longer treatment times (Figure 4B). These vacuoles eventually merged into larger vacuoles, and this accumulation led to cell death, suggesting that tramadol may have a cytotoxic effect on breast cancer cells. Similar observations have been reported in human hepatocellular carcinoma, where sorafenib induces ER dilation, UPR pathway activation, and cytoplasmic vacuolation associated with nonapoptotic cell death [45].

To further examine these vacuoles, we utilized transmission electron microscopy (TEM) to observe their morphology at higher magnification (selection by yellow squares). In tramadol-treated MDA-MB-231 and MCF-7 cells, there were numerous vacuoles (Figure 4C). These included autophagic vacuoles containing intracellular material (indicated by blue arrows) and empty lipid droplets (indicated by green LD). Under ER stress, an enlarged ER membrane (indicated by pink ER) contributes to autophagosome formation. The mitochondria (shown as yellow M) in the control cells maintained a fusion state, whereas in the tramadol-treated cells, most of the mitochondria were in a fission state.

**Figure 5.**
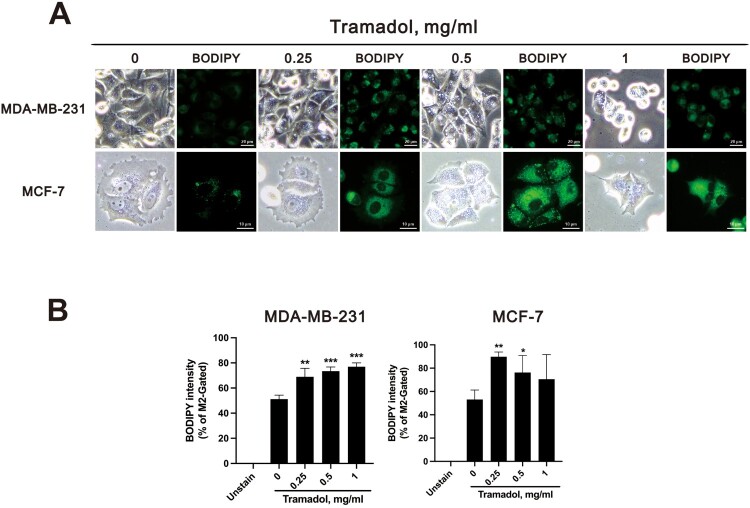
Lipid droplet accumulation during tramadol-induced cell death in breast cancer cells. A MDA-MB-231 and MCF-7 cells treated with tramadol were stained with BODIPY to visualize lipid droplets via fluorescence microscopy; B the results were quantified via flow cytometry. Statistical analysis was performed using one-way ANOVA with Tukey’s multiple comparisons (*p < 0.05, **p < 0.01, ***p < 0.001 vs. control).

To further assess lipid droplets, we employed BODIPY staining to visualize their distribution. The results revealed an increase in lipid droplets in response to 0.25 and 0.5 mg/ml tramadol, with a punctate distribution within the cells (Figure 5A). At 1 mg/ml, however, the lipid droplets became more uniformly distributed throughout the cells. Flow cytometry validated these findings, indicating that tramadol significantly increased lipid droplet accumulation in both MDA-MB-231 and MCF-7 cells (Figure 5B).

### Tramadol induced autophagy in breast cancer cells

**Figure 6.**
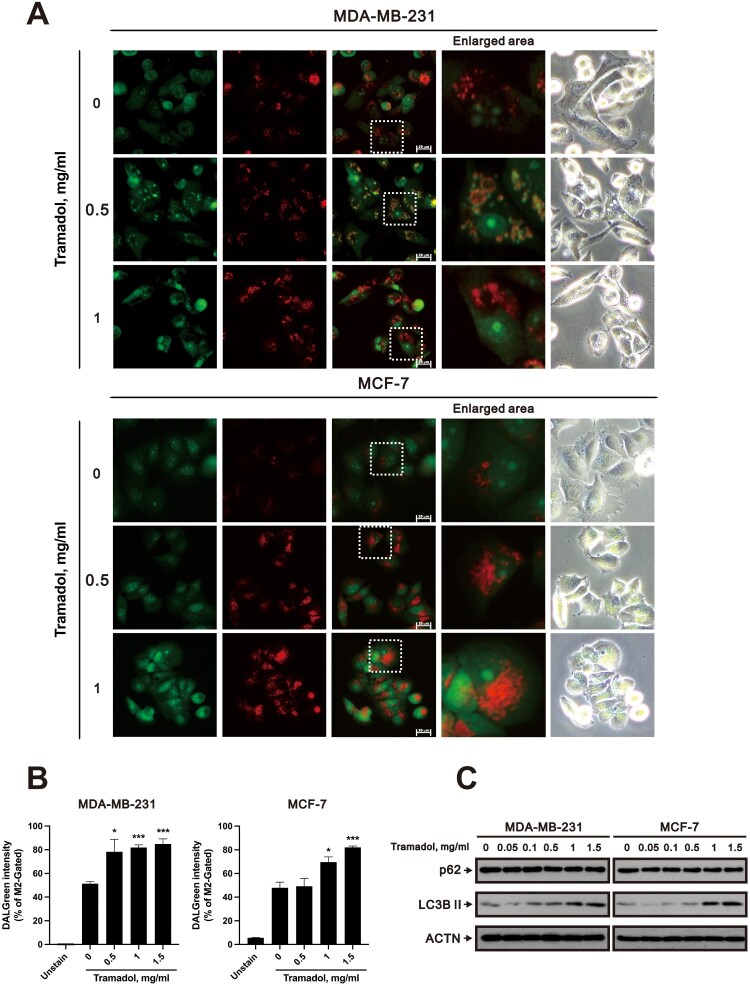
Effects of tramadol on autophagy in MDA-MB-231 and MCF-7 cells. A Fluorescence microscopy images of MDA-MB-231 and MCF-7 cells treated with 0, 0.5, or 1 mg/ml tramadol were captured after staining with AO. In these images, viable cells fluoresce green, whereas acidic compartments, such as those associated with autophagic or lysosomal activity, exhibit a red to orange hue. Squares indicate representative regions shown as enlarged areas in the fluorescent images. B MDA-MB-231 and MCF-7 cells were treated with 0, 0.5, 1, or 1.5 mg/mL tramadol and then stained with DALGreen to analyze autophagy; autophagy was quantified by flow cytometry. Statistical analysis was performed using one-way ANOVA with Tukey’s multiple comparisons (*p < 0.05, ***p < 0.001 vs. control). C MDA-MB-231 and MCF-7 cells were treated with 0, 0.05, 0.1, 0.5, 1, or 1.5 mg/ml tramadol. ACTN was used as a loading control.

Oxidative stress and ER stress are significant contributors to cell death, often resulting from mitochondrial permeability transition and impaired autophagy [46]. Autophagy is a highly conserved cellular process that involves the degradation and recycling of cellular components, including damaged proteins and organelles [47]. This process is essential for maintaining cellular homeostasis and promoting cell survival under stress conditions. During autophagy, cytoplasmic components are sequestered into double-membraned structures called autophagosomes, which subsequently fuse with lysosomes for degradation [48]. Acridine orange (AO) is a cell-permeable green fluorophore that can be protonated and trapped in acidic vesicular organelles (AVOs). Under AO staining, the cytoplasm and nucleoli fluoresce green, whereas acidic compartments, such as lysosomes or autophagolysosomes, fluoresce bright red or orange red with blue-light excitation. To investigate whether tramadol induces autophagy in breast cancer cells, we conducted AO staining and analyzed the fluorescence images presented in Figure 6A. The results revealed that in both MDA-MB-231 and MCF-7 cells, the red fluorescence representing AVOs (squares indicate representative regions shown as enlarged areas) increased in both size and quantity as the tramadol dose increased. This observation suggested that treatment with tramadol enhanced the formation of acidic vesicles, indicating increased autophagic activity.

In addition to AO staining, we utilized DALGreen, a dye that selectively integrates into the autophagosome membrane upon autophagosome formation, in conjunction with flow cytometry to assess autophagy in live cells. This approach allows for the quantitative measurement of autophagic activity by analyzing the fluorescence intensity associated with autophagosomes. The results indicated that treatment with tramadol significantly enhanced autophagic activity in both MDA-MB-231 and MCF-7 cells in a dose-dependent manner (Figure 6B). LC3-I is converted into LC3-II during autophagy; LC3-II is associated with the autophagosomal membrane and is considered a crucial marker of autophagy [49]. To elucidate the effects of tramadol on autophagy, we performed Western blotting to assess the protein expression levels of LC3B. The results indicated that treatment with tramadol significantly induced LC3B II protein expression in both MDA-MB-231 and MCF-7 cells (Figure 6C). Collectively, these findings provide compelling evidence that tramadol induces autophagy in breast cancer cells.

### Tramadol induced mitochondrial dysfunction in MDA-MB-231 and MCF-7 cells

**Figure 7.**
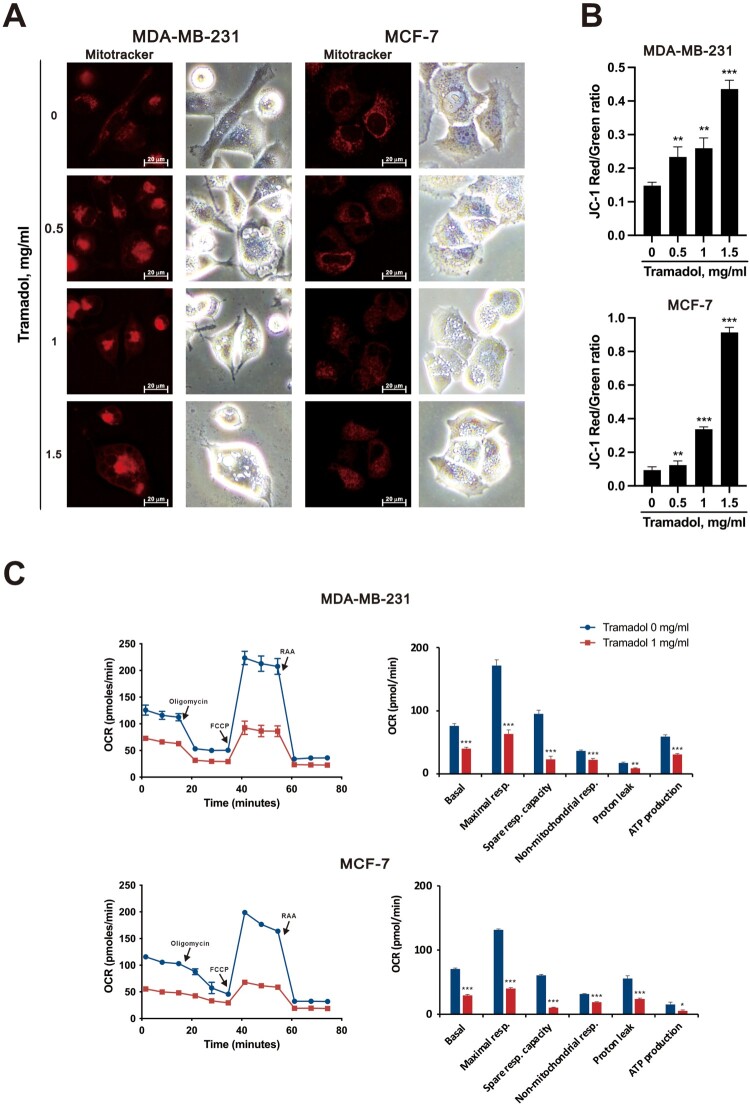
Effects of tramadol on mitochondrial function in MDA-MB-231 and MCF-7 cells. A Fluorescence microscopy images of MDA-MB-231 and MCF-7 cells treated with 0, 0.5, 1, or 1.5 mg/ml tramadol were captured after staining with MitoTracker. B MDA-MB-231 and MCF-7 cells were treated with 0, 0.5, 1, or 1.5 mg/mL tramadol and then stained with JC-1 to assess the mitochondrial membrane potential; mitochondrial membrane potential was quantified via flow cytometry. Statistical analysis was performed using one-way ANOVA with Tukey’s multiple comparisons (**p < 0.01, ***p < 0.001 vs. control). C MDA-MB-231 and MCF-7 cells were treated with 1 mg/mL tramadol, and the OCR was assessed using a Seahorse XF24 analyzer. Data are shown as mean ± SEM; *p < 0.05, **p < 0.01, ***p < 0.001 vs. control (two-tailed unpaired Student’s t-test).

The morphology of mitochondria is regulated primarily by the processes of fusion and fission, which are essential for maintaining mitochondrial function and ensuring strict quality control. To investigate whether tramadol affects mitochondrial morphology, we used MitoTracker to label mitochondria. Among MDA-MB-231 cells, those in the control group presented a tubular distribution of mitochondria throughout the cell; however, following treatment with tramadol, the mitochondria clustered together, emitting stronger red fluorescence (Figure 7A). In MCF-7 cells, the control group also exhibited a tubular distribution of mitochondria; however, after treatment with tramadol, the distribution of mitochondria was less widespread. These findings indicated that tramadol significantly altered mitochondrial morphology in breast cancer cells.

To investigate the effects of tramadol on the mitochondrial membrane potential in breast cancer cells, JC-1 dye was used. This dye is particularly useful because it can indicate changes in the mitochondrial membrane potential, specifically highlighting the phenomenon of mitochondrial membrane potential loss. Our results revealed that in both MDA-MB-231 and MCF-7 cells, the loss of the mitochondrial membrane potential increased in a dose-dependent manner with increasing doses of tramadol (Figure 7B). These findings suggest that tramadol not only affects mitochondrial morphology but also disrupts mitochondrial membrane potential, a critical aspect of mitochondrial function. Next, we employed the Seahorse XF Cell Mito Stress Test to assess mitochondrial bioenergetic function more comprehensively. This assay allows the real-time measurement of key parameters of mitochondrial bioenergetic functions, including the oxygen consumption rate (OCR), basal respiration, ATP production, and maximal respiratory capacity of cells. The results from this analysis confirmed that treatment with tramadol significantly impaired mitochondrial OCR (Figure 7C) and decreased basal respiration, maximal respiration, spare respiratory capacity, nonmitochondrial respiration, proton leakage, and ATP production in both MDA-MB-231 and MCF-7 cells (Figure 7C). Collectively, these findings indicate that tramadol induces both morphological and functional changes in mitochondria, which may contribute to its cytotoxic effects on breast cancer cells.

### Tramadol induced paraptosis in MDA-MB-231 and MCF-7 cells

**Figure 8.**
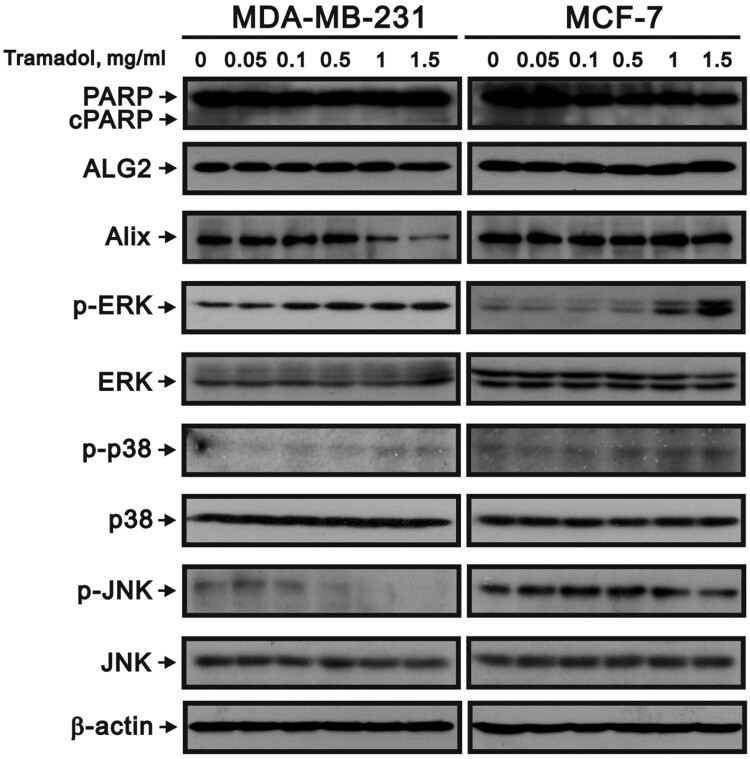
Tramadol influenced cell death pathways and paraptosis-associated signaling in breast cancer cells. MDA-MB-231 and MCF-7 cells were treated with 0, 0.05, 0.1, 0.5, 1, or 1.5 mg/ml tramadol. β-Actin was used as a loading control.

Cell death pathways are classified on the basis of their signaling dependencies, morphological characteristics, and molecular mechanisms. Our data revealed that tramadol might induce paraptosis-like cell death in breast cancer cells, as indicated by distinct cellular responses, such as ER stress, ROS generation, autophagy, mitochondrial dysfunction, and extensive vacuole formation. Notably, paraptosis represents a distinct, nonapoptotic form of PCD that occurs independently of caspase activation. Mitogen-activated protein kinase (MAPK) components, i.e. p38 MAPK, JNK (c-Jun N-terminal kinase), and ERK (extracellular signal-regulated kinase), play critical roles in mediating stress responses within the cell, and their abnormal activation is essential for driving paraptotic cell death. The first natural inhibitor of paraptosis identified was ALG-2-interacting protein X (Alix), a protein that was initially cloned from apoptosis-linked gene 2 (ALG-2), a calcium-binding protein involved in T cell receptor-induced cell death [50]. To investigate the molecular changes induced by tramadol, we treated both MDA-MB-231 and MCF-7 cells with tramadol followed by Western blot analysis. In the context of caspase-dependent apoptosis, poly(ADP–ribose) polymerase (PARP) is cleaved by caspase 3, a hallmark of this apoptotic pathway. Our results revealed that tramadol did not induce PARP cleavage, which suggests that tramadol does not activate the caspase cascade, indicating a lack of apoptotic signaling through this pathway (Figure 8). Additionally, our analysis revealed that tramadol activated the p38 and ERK signaling pathways in both MDA-MB-231 and MCF-7 cells. Moreover, we observed a significant decrease in Alix protein expression, specifically in MDA-MB-231 cells, following treatment with tramadol.

### Tramadol-induced morphological changes in MDA-MB-231 cells with HIF-1α, ATF4, or PDCD6IP knockouts

**Figure 9.**
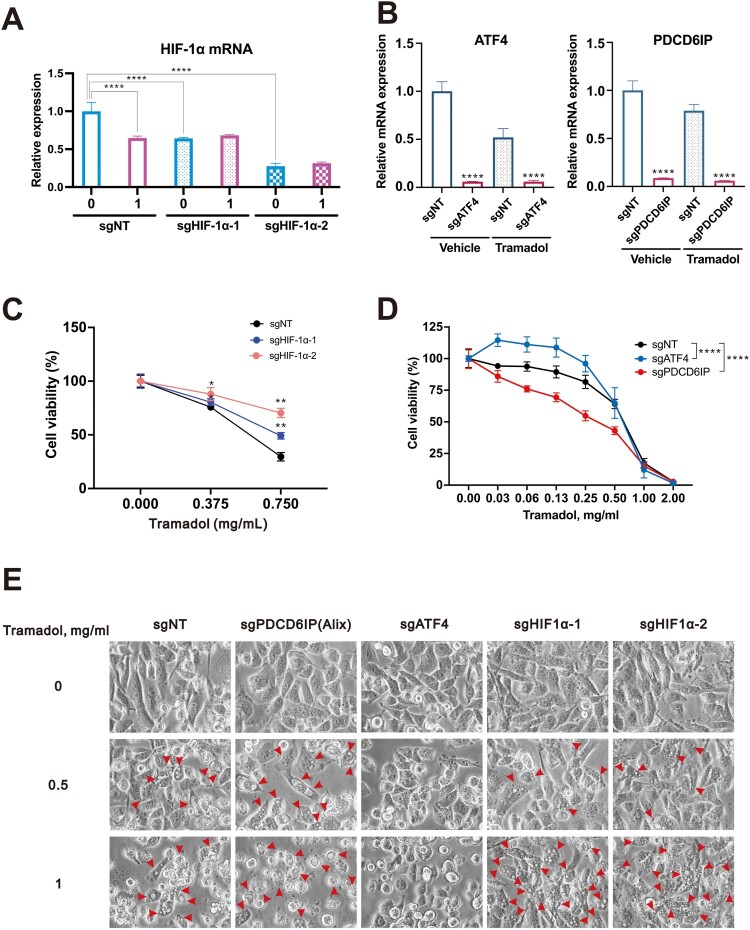
Functional roles of HIF-1α, ATF4, and Alix in tramadol-induced cytotoxicity and morphological alterations in breast cancer cells. A-Β After HIF-1α, ATF4, and PDCD6IP were knocked out in MDA-MB-231 cells using the CRISPR-Cas9 system, the knockout efficiency at the mRNA level was assessed via qPCR. Data are shown as mean ± SEM; ****p < 0.0001 vs. control (two-tailed unpaired Student’s t-test). C MDA-MB-231 cells were treated with 0.375 or 0.75 mg/ml tramadol for 24 h. Cell viability was determined by CellTiter-Glo assay. Two-way ANOVA with Dunnett’s multiple comparisons was performed, and the results were compared with the vehicle group. *p < 0.05, **p < 0.01. D MDA-MB-231 cells were treated with 0, 0.03, 0.06, 0.13, 0.25, 0.5, 1, 2 mg/ml tramadol for 24 h. Cell viability was determined by CellTiter-Glo assay. Two-way ANOVA with Dunnett’s multiple comparisons was performed, and the results were compared with the vehicle group. ****p < 0.0001. E MDA-MB-231 cells were exposed to tramadol (0, 0.5, or 1 mg/mL) for 4 h, and morphological alterations were examined using phase-contrast microscopy. Arrows indicate cytoplasmic vacuoles.

Finally, we conducted knockout experiments targeting the *HIF-1α*, *ATF4* and the *programmed cell death 6 interacting protein* (*PDCD6IP*) gene, which encodes the Alix protein, to investigate their roles in tramadol-induced cell death in breast cancer cells. To investigate the functional contribution of ATF4, HIF-1α, and Alix to tramadol-mediated cytotoxic effects, we analyzed the morphological outcomes in MDA-MB-231 cells following gene-specific knockout using CRISPR/Cas9 technology. Successful knockout of *HIF-1α*, *ATF4*, and *PDCD6IP* (Alix) was confirmed by qPCR, which showed markedly reduced mRNA expression levels, indicating efficient gene disruption (Figure 9A-B). To assess the role of HIF-1α in tramadol-induced cytotoxicity, we measured cell viability following tramadol treatment in *HIF-1α* knockout cells. At 750 µg/mL tramadol, both sgHIF-1α constructs conferred protection against cell death, with sgHIF-1α−2 exhibiting a more substantial protective effect than sgHIF-1α−1 (Figure 9C), underscoring the involvement of HIF-1α in the cellular stress response to tramadol. Notably, *ATF4* knockout also markedly attenuated tramadol-induced cytotoxicity in MDA-MB-231 cells, suggesting that ATF4 is a key mediator of tramadol’s cell-killing effects (Figure 9D). In contrast, deletion of *PDCD6IP* enhanced the cytotoxicity of tramadol, indicating that Alix may function as a protective factor under tramadol-induced stress.

Upon tramadol exposure, control cells expressing non-targeting sgRNA (sgNT) exhibited a dose-dependent increase in cytoplasmic vacuole formation and cell enlargement (Figure 9E). In *ATF4*-deficient cells (sgATF4), the extent of vacuolization was notably diminished, implicating ATF4 as a key driver of this phenotype. Conversely, *HIF-1α* knockout cells (sgHIF1α−1 and sgHIF1α−2) displayed vacuolization levels similar to those of control cells; however, these cells maintained a more intact morphology with fewer instances of detachment and rounding. This suggests that while HIF-1α may not directly influence vacuole formation, it plays a protective role in sustaining cell integrity under tramadol-induced stress. Interestingly, loss of *PDCD6IP* resulted in pronounced morphological deterioration following tramadol treatment. Cells lacking Alix exhibited extensive rounding and detachment, indicating heightened vulnerability to tramadol-induced cytotoxicity and suggesting a protective function for Alix in this context. Together, these findings suggest that ATF4 facilitates cytoplasmic vacuolization (red arrows), HIF-1α contributes to cellular resilience, and Alix functions as a negative regulator of tramadol-induced cell death in MDA-MB-231 cells.

## Discussion

This study uncovered that tramadol impaired cancer cell viability through two mechanistically linked but separate processes: the stabilization of HIF-1α under normal oxygen conditions and the activation of paraptotic cell death driven by oxidative stress and ER dysfunction. These findings shed light on previously unrecognized cellular effects of tramadol, suggesting potential applications beyond its traditional use as a pain reliever. In cancer treatment, the resistance of cancer cells to apoptosis poses a significant challenge and contributes to numerous cancer-related deaths. A promising strategy to overcome this resistance involves targeting cancer cells through the induction of paraptosis, a recently identified alternative caspase-independent cell death pathway. This study elucidated the molecular mechanisms by which tramadol exerts its anticancer effects and induces paraptosis, a nonapoptotic form of programmed cell death, in MDA-MB-231 and MCF-7 breast cancer cells. Paraptosis induced by tramadol involves the coordinated activation of ROS, hypoxia signaling, and ER stress, all converging on the p-eIF2α/ATF4/CHOP axis. In parallel, autophagy disruption, mitochondrial impairment, and lipid droplet buildup contribute to extensive cytoplasmic vacuolization, a defining feature of paraptotic cell death.

Paraptosis is a distinct type of nonapoptotic cell death characterized by dilation of the ER and/or mitochondria. Unlike apoptotic cells, paraptotic cells do not exhibit features such as cell shrinkage, DNA fragmentation, or caspase activation. Instead, paraptosis involves disruptions in cellular protein and ion balance, particularly concerning calcium ion transport [24]. This form of cell death can be triggered by various pharmacological agents, including natural compounds, metal complexes, proteasome inhibitors, and specific ion regulators [51, 52]. Key pathways that initiate paraptosis include MAPK activation via insulin-like growth factor-I receptor (IGFIR), ER stress resulting from proteasome inhibition or other factors, osmotic stress due to ROS production, and impaired mitochondrial function [53]. The literature also indicates that downregulation of the phosphorylation of MAPK signaling pathways inhibits tumor bone metastasis and osteoclastogenesis in breast cancer, suggesting that MAPK signaling plays a critical role not only in paraptosis but also in cancer progression and metastatic regulation [54]. Furthermore, imbalances in ion homeostasis can lead to ER and mitochondrial swelling. In contrast, certain genes, such as *PDCD6IP*, *heme transporter 1* (*FLVCR1*), and *thioredoxin reductase 1* (*TXNRD1*), are known to inhibit paraptosis [50, 51, 55]. Although apoptosis and paraptosis were initially thought to function independently, emerging research suggests a potential connection between the two, with the tumor proteins p53 (TP53) and MAPK acting as key mediators. In situations where TP53 is deficient, MAPK-driven apoptosis and paraptosis may interact in either a synergistic or competitive manner. For example, fusaric acid, a mycotoxin found in food, can induce either apoptosis or paraptosis in a dose-dependent manner in human peripheral blood mononuclear cells and THP-1 cells [56]. While paraptosis is generally considered a caspase-independent form of regulated cell death, caspase 9 has been implicated in regulating both apoptosis and paraptosis, with paraptotic cell death becoming more pronounced when apoptosis is inhibited [57]. Therefore, targeting paraptosis represents a promising strategy for overcoming the resistance to apoptosis commonly observed in cancer cells.

Our previous study primarily investigated the interaction between tramadol and doxorubicin [19]; however, the possibility that tramadol may also modulate the cytotoxic effects of other chemotherapeutic drugs remains an important direction for future research. Given tramadol’s pleiotropic mechanisms – including ROS generation, ER stress induction, and modulation of HIF-1α and ATF4 signaling – it is plausible that its impact on drug efficacy could vary according to the molecular mechanisms of individual chemotherapeutic agents. For example, drugs such as paclitaxel and cisplatin, which also induce oxidative and ER stress responses, might exhibit additive or synergistic effects when combined with tramadol. In contrast, agents that act primarily through DNA intercalation or topoisomerase inhibition may display distinct patterns of interaction. Potential pharmacokinetic interference should also be taken into account, as tramadol metabolism via CYP2D6 could influence systemic drug exposure or toxicity profiles. Furthermore, since our findings were derived from *in vitro* experiments in breast cancer cell lines, *in vivo* pharmacodynamic interactions and tumor microenvironmental influences remain to be elucidated. From a translational standpoint, the identification of tramadol as a modulator of the HIF-1α/ATF4 axis provides new insights into therapeutic strategies targeting stress-adaptive pathways in resistant breast cancer subtypes. The dual involvement of HIF-1α stabilization and ATF4 activation suggests that tramadol may exploit the cellular stress network to drive nonapoptotic paraptotic death, offering a potential route to overcome apoptosis resistance commonly observed in TNBC. Pharmacological or genetic inhibition of this adaptive axis has been proposed to restore drug sensitivity in hypoxic tumors [58], and our findings imply that tramadol or its derivatives might achieve similar outcomes through partial pathway modulation. Future studies integrating tramadol with targeted inhibitors or conventional chemotherapeutics could clarify whether these effects translate into improved therapeutic efficacy *in vivo*.

Tramadol differentially affected three primary UPR pathways – IRE1, ATF6, and PERK – in MDA-MB-231 and MCF-7 breast cancer cells. Specifically, tramadol induced the expression of the IRE1, ATF6, and PERK proteins in MCF-7 cells but decreased their expression in MDA-MB-231 cells. However, the expression of components of the p-eIF2α/ATF4/CHOP signaling pathway, which is activated by the UPR, was observed in both cell types. MCF-7 and MDA-MB-231 cells are both invasive ductal breast carcinoma cells but differ significantly in their phenotypic and genotypic profiles. MCF-7 cells are hormone dependent, estrogen receptor positive, progesterone receptor positive, and HER2 negative, whereas MDA-MB-231 cells are triple negative. The absence of hormone receptors in MDA-MB-231 cells renders them unresponsive to antiestrogen and targeted therapies, such as tamoxifen or anti-HER2 therapies [59–61]. In terms of metabolism, MCF-7 cells exhibit a Pasteur-type profile, primarily generating ATP through oxidative phosphorylation under normoxic conditions but increasing glycolytic activity under hypoxic conditions [62]. In contrast, MDA-MB-231 cells follow a Warburg-type metabolism, relying mainly on glycolysis for ATP production in both normoxic and hypoxic environments [63]. Additionally, MCF-7 cells display an epithelial phenotype, whereas MDA-MB-231 cells have a more mesenchymal phenotype and are known for their multidrug resistance [64]. Owing to their high metastatic potential and lack of specific target receptors, MDA-MB-231 cells represent a challenging subtype of TNBC. As a result, various candidate small-molecule drugs, including both single-target and repurposed agents, have emerged as promising therapies for TNBC [59, 60]. The results of this study revealed that tramadol may induce paraptotic cell death in both MDA-MB-231 and MCF-7 cells through distinct pathways. The development of paraptosis inducers could open new avenues for TNBC treatment.

In cancer, HIFs have been described as both oncogenes and tumor suppressors, depending on the cellular and environmental context [37, 65, 66]. Studies have shown that the transcriptional response of HIF-1α under hypoxic conditions is driven by epigenetic regulation at low oxygen levels and can promote high-risk tumor characteristics. Interestingly, HIF-1α targets expressed in both normoxic and hypoxic regions may offer new therapeutic opportunities for eradicating solid tumors [67]. In our study, however, tramadol-induced HIF-1α stabilization under normoxic conditions did not enhance tumor aggressiveness. Instead, tramadol-treated cells showed reduced migratory and metastatic potential [19], implying that HIF-1α activation alone is insufficient to drive tumor progression and may be counterbalanced by other tramadol-mediated effects, such as cell-cycle arrest and apoptosis induction. Consistent with this, tramadol increased the sub-G1 population and promoted apoptosis. Interestingly, HIF-1α knockout cells displayed higher sub-G1 levels but fewer late apoptotic cells, suggesting that HIF-1α contributes to the proper execution of cell death following tramadol treatment. Recent studies have shown that hypoxic tumor microenvironments markedly reduce cisplatin sensitivity through HIF-1α – dependent signaling and disrupted redox homeostasis [68]. In contrast, our findings indicate that tramadol-induced stabilization of HIF-1α under normoxia may instead enhance doxorubicin sensitivity. This apparent paradox highlights the context-dependent nature of HIF-1α regulation: while hypoxia-driven activation typically promotes chemoresistance, tramadol appears to rewire HIF-1α and redox signaling to favor cell death rather than survival. Thus, tramadol may mimic or partially activate elements of the hypoxic response in breast cancer cells, eliciting stress-adaptive mechanisms that culminate in cytotoxicity rather than protection. These findings underscore the complex and dual roles of HIF-1α in cancer biology and emphasize the need to further investigate how tramadol modulates HIF-1α – dependent pathways. This highlights the importance of considering additional regulatory mechanisms of HIFs that influence tramadol’s overall impact on breast cancer biology.

Notably, TNBC exhibits a more pronounced hypoxic signature compared to other breast cancer subtypes [69]. TNBC cells, through the production of inflammatory mediators and adaptation to hypoxia, are capable of regulating HIF-1α activity [70]. As a highly hypoxic solid tumor, TNBC is characterized by a tumor microenvironment that is driven by elevated oxygen consumption from tumor cells, coupled with insufficient vascularization, which exacerbates the hypoxic conditions. Acute and chronic inflammation further exacerbate this condition, leading to the generation of NO and ROS within TNBC cells [71]. These factors work collectively to maintain the activity and stability of HIF-1α, a critical mediator of cellular adaptation to hypoxia. Consequently, the persistent hypoxic microenvironment and HIF-1α stabilization significantly contribute to the aggressive nature and therapeutic resistance of TNBC, underscoring the need for targeted strategies to mitigate hypoxia-driven pathways in this challenging breast cancer subtype. However, our current findings reveal that the complex involvement of HIF-1α in tramadol-induced cytotoxicity, cell cycle progression, and morphological adaptations. Our data showed that tramadol-induced cytotoxicity, subG1 populations, and apoptosis were dependent on HIF-1α stability in MDA-MB-231 cells. Given this potential tumor-suppressive function of HIF-1α in normoxic TNBC cells, caution should be exercised when developing therapeutic strategies aimed at inhibiting HIF-1α signaling.

In conclusion, this study delineated the molecular basis of tramadol’s antitumor activity, demonstrating its ability to trigger paraptosis – a non-apoptotic form of programmed cell death – in breast cancer cells. Tramadol-induced paraptosis is orchestrated by elevated ROS levels and endoplasmic reticulum stress, converging on the p-eIF2α/ATF4/CHOP signaling cascade. Notably, tramadol also stabilizes HIF-1α under normoxic conditions, further contributing to the cellular stress response. Concurrent alterations, including impaired autophagy, mitochondrial dysfunction, and intracellular lipid droplet accumulation, collectively drive the formation of prominent cytoplasmic vacuoles, a hallmark of paraptotic cell death. These findings uncover previously unrecognized anticancer mechanisms of tramadol and suggest its potential therapeutic utility distinct from conventional apoptosis-based treatments.

## Authors’ contribution

Zih-Syuan Wu: Conceptualization, Data Curation, Validation, Formal analysis, Writing – Original Draft. Shih-Ming Huang: Conceptualization, Methodology, Project administration, Funding acquisition, Writing – Original Draft. Yi-Hsuan Huang: Conceptualization, Data Curation, Validation, Formal analysis, Funding acquisition, Writing – Original Draft, Writing – Review & Editing. All the authors have read and approved the final manuscript.

## Data Availability

The datasets generated and/or analyzed during this study are available from the corresponding author upon reasonable request. Additionally, our RNA-seq data has been deposited in the NCBI Gene Expression Omnibus under accession number GSE282008.

## References

[1] Miller KD, Nogueira L, Devasia T, et al. Cancer treatment and survivorship statistics, 2022. CA Cancer J Clin. 2022;72(5):409–436. 35736631 10.3322/caac.21731

[2] Siegel RL, Miller KD, Jemal A. Cancer statistics. CA Cancer J Clin. 2019;69(1):7–34. 30620402 10.3322/caac.21551

[3] De Groef A, Meeus M, Heathcote LC, et al. Treating persistent pain after breast cancer: practice gaps and future directions. J Cancer Surviv. 2023;17(6):1698–1707. doi:10.1007/s11764-022-01194-z 35275361 PMC8914454

[4] Manfuku M, Nishigami T, Mibu A, et al. Comparison of central sensitization-related symptoms and health-related quality of life between breast cancer survivors with and without chronic pain and healthy controls. Breast Cancer. 2019;26(6):758–765. doi:10.1007/s12282-019-00979-y 31127501

[5] Wang WL, Hao YH, Pang X, et al. Cancer pain: molecular mechanisms and management. Mol Biomed. 2025;6(1):45. doi:10.1186/s43556-025-00289-0 40579593 PMC12205135

[6] Ramirez MF, Strang A, Roland G, et al. Perioperative pain management and cancer outcomes: A narrative review. J Pain Res. 2023;16:4181–4189. doi:10.2147/JPR.S432444 38078017 PMC10710188

[7] Szczepaniak A, Fichna J, Zielinska M. Opioids in cancer development, progression and metastasis: focus on colorectal cancer. Curr Treat Options Oncol. 2020;21(1):6. doi:10.1007/s11864-019-0699-1 31970561 PMC6976545

[8] Wang R, Li S, Wang B, et al. Impact of opioids and mu-opioid receptors on oncologic metastasis. Am J Cancer Res. 2024;14(9):4236–4247. doi:10.62347/SCLS3277 39417177 PMC11477826

[9] Weingaertner IR, Koutnik S, Ammer H. Chronic morphine treatment attenuates cell growth of human BT474 breast cancer cells by rearrangement of the ErbB signalling network. PLoS One. 2013;8(1):e53510. doi:10.1371/journal.pone.0053510 23308242 PMC3538590

[10] Lucia M, Luca T, Federica DP, et al. Opioids and breast cancer recurrence: A systematic review. Cancers (Basel). 2021;13(21):5499. doi:10.3390/cancers13215499 34771662 PMC8583615

[11] Montagna G, Gupta HV, Hannum M, et al. Intraoperative opioids are associated with improved recurrence-free survival in triple-negative breast cancer. BJA: British Journal of Anaesthesia. 2020;126(2):367. doi:10.1016/j.bja.2020.10.021 33220939 PMC8014943

[12] Kim MH, Oh JE, Park S, et al. Tramadol use is associated with enhanced postoperative outcomes in breast cancer patients: a retrospective clinical study with in vitro confirmation. Br J Anaesth. 2019;123(6):865–876. doi:10.1016/j.bja.2019.09.004 31591020

[13] Kampe S, Wolter K, Warm M, et al. Clinical equivalence of controlled-release oxycodone 20 mg and controlled-release tramadol 200 mg after surgery for breast cancer. Pharmacology. 2009;84(5):276–281. doi:10.1159/000242998 19797937

[14] Besic N, Smrekar J, Strazisar B. Acute pain and side effects after tramadol in breast cancer patients: results of a prospective double-blind randomized study. Sci Rep. 2020;10(1):18766. doi:10.1038/s41598-020-75961-2. PubMed PMID: 33127945; PubMed Central PMCID: PMC7599328 04/2015-doretaonko/si from the pharmaceutical company Krka d.d. Novo mesto. J. Smrekar has received a honorarium for the statistical analysis of our study from the pharmaceutical company Krka d.d. Novo mesto. 33127945 PMC7599328

[15] Edinoff AN, Kaplan LA, Khan S, et al. Full opioid agonists and tramadol: pharmacological and clinical considerations. Anesth Pain Med. 2021;11(4):e119156. 34692448 10.5812/aapm.119156 PMC8520671

[16] Grond S, Sablotzki A. Clinical pharmacology of tramadol. Clin Pharmacokinet. 2004;43(13):879–923. doi:10.2165/00003088-200443130-00004 15509185

[17] Xia M, Tong J-H, Zhou Z-Q, et al. Tramadol inhibits proliferation, migration and invasion via α2-adrenoceptor signaling in breast cancer cells. European Review for Medical & Pharmacological Sciences. 2016;20(1):157–165. 26813469

[18] Kim MH, Lee J-R, Kim K-J, et al. Identification for antitumor effects of tramadol in a xenograft mouse model using orthotopic breast cancer cells. Sci Rep. 2021;11(1):22113. doi:10.1038/s41598-021-01701-9 34764420 PMC8586351

[19] Huang YH, Sue SH, Wu ZS, et al. Antitumorigenic effect of tramadol and synergistic effect With doxorubicin in human breast cancer cells. Front Oncol. 2022;12:811716. doi:10.3389/fonc.2022.811716 35155248 PMC8826738

[20] Liu LC, Wu ZS, Chen JL, et al. Mitochondrial dysfunction involved in the cytotoxicity of tramadol in human endometrial carcinoma cells. Int J Mol Sci. 2022;24(1):99. doi:10.3390/ijms24010099 36613541 PMC9820256

[21] Neophytou CM, Trougakos IP, Erin N, et al. Apoptosis deregulation and the development of cancer multi-drug resistance. Cancers (Basel). 2021;13(17):4363. doi:10.3390/cancers13174363 34503172 PMC8430856

[22] Safa AR. Drug and apoptosis resistance in cancer stem cells: a puzzle with many pieces. Cancer Drug Resist. 2022;5(4):850–872. doi:10.20517/cdr.2022.20 36627897 PMC9771762

[23] Dandoti S. Mechanisms adopted by cancer cells to escape apoptosis – A review. Biocell. 2021;45(4):863–884. doi:10.32604/biocell.2021.013993

[24] Hanson S, Dharan A, VJ P, et al. Paraptosis: a unique cell death mode for targeting cancer. Front Pharmacol. 2023;14:1159409. doi:10.3389/fphar.2023.1159409 37397502 PMC10308048

[25] Nguyen PL, Lee CH, Lee H, et al. Induction of paraptotic cell death in breast cancer cells by a novel pyrazolo[3,4-h]quinoline derivative through ROS production and endoplasmic reticulum stress. Antioxidants (Basel). 2022;11(1):117. doi:10.3390/antiox11010117 35052621 PMC8773266

[26] Fontana F, Raimondi M, Marzagalli M, et al. The emerging role of paraptosis in tumor cell biology: perspectives for cancer prevention and therapy with natural compounds. Biochim Biophys Acta Rev Cancer. 2020;1873(2):188338. doi:10.1016/j.bbcan.2020.188338 31904399

[27] Chen X, Shi C, He M, et al. Endoplasmic reticulum stress: molecular mechanism and therapeutic targets. Signal Transduction and Targeted Therapy. 2023;8(1):352. doi:10.1038/s41392-023-01570-w 37709773 PMC10502142

[28] Walter P, Ron D. The unfolded protein response: from stress pathway to homeostatic regulation. Science. 2011;334(6059):1081–1086. doi:10.1126/science.1209038 22116877

[29] Khanna M, Agrawal N, Chandra R, et al. Targeting unfolded protein response: a new horizon for disease control. Expert Rev Mol Med. 2021;23. 10.1017/erm.2021.2 33660595

[30] Yamamoto K, Yoshida H, Kokame K, et al. Differential contributions of ATF6 and XBP1 to the activation of endoplasmic reticulum stress-responsive cis-acting elements ERSE, UPRE and ERSE-II. J Biochem. 2004;136(3):343–350. doi:10.1093/jb/mvh122 15598891

[31] Huang S, Xing Y, Liu Y. Emerging roles for the ER stress sensor IRE1α in metabolic regulation and disease. J Biol Chem. 2019;294(49):18726–18741. doi:10.1074/jbc.REV119.007036. 31666338 PMC6901316

[32] Donnelly N, Gorman AM, Gupta S, et al. The eIF2α kinases: their structures and functions. Cell Mol Life Sci. 2013;70(19):3493–3511. doi:10.1007/s00018-012-1252-6 23354059 PMC11113696

[33] Chen Q, Samidurai A, Thompson J, et al. Endoplasmic reticulum stress-mediated mitochondrial dysfunction in aged hearts. Biochimica et Biophysica Acta (BBA) – Molecular Basis of Disease. 2020;1866(11):165899. doi:10.1016/j.bbadis.2020.165899. 32698045

[34] Chen Q, Thompson J, Hu Y, et al. Metformin attenuates ER stress – induced mitochondrial dysfunction. Transl Res. 2017;190:40–50. doi:10.1016/j.trsl.2017.09.003. 29040818 PMC5705457

[35] Bronner Denise N, Abuaita Basel H, Chen X, et al. Endoplasmic reticulum stress activates the inflammasome via NLRP3 – and caspase-2-driven mitochondrial damage. Immunity. 2015;43(3):451–462. doi:10.1016/j.immuni.2015.08.008 26341399 PMC4582788

[36] Semenza GL, Wang GL. A nuclear factor induced by hypoxia via de novo protein synthesis binds to the human erythropoietin gene enhancer at a site required for transcriptional activation. Mol Cell Biol. 1992;12(12):5447–5454. 1448077 10.1128/mcb.12.12.5447-5454.1992 PMC360482

[37] Semenza GL. Oxygen sensing, hypoxia-inducible factors, and disease pathophysiology. Annu Rev Pathol. 2014;9:47–71. doi:10.1146/annurev-pathol-012513-104720 23937437

[38] Bruick RK, McKnight SL. A conserved family of prolyl-4-hydroxylases that modify HIF. Science. 2001;294(5545):1337–1340. doi:10.1126/science.1066373 11598268

[39] Maxwell PH, Wiesener MS, Chang GW, et al. The tumour suppressor protein VHL targets hypoxia-inducible factors for oxygen-dependent proteolysis. Nature. 1999;399(6733):271–275. doi:10.1038/20459 10353251

[40] Semenza GL. Targeting HIF-1 for cancer therapy. Nat Rev Cancer. 2003;3(10):721–732. doi:10.1038/nrc1187 13130303

[41] Beasley NJ, Leek R, Alam M, et al. Hypoxia-inducible factors HIF-1alpha and HIF-2alpha in head and neck cancer: relationship to tumor biology and treatment outcome in surgically resected patients. Cancer Res. 2002;62(9):2493–2497. 11980639

[42] Sarcinelli C, Dragic H, Piecyk M, et al. ATF4-Dependent NRF2 transcriptional regulation promotes antioxidant protection during endoplasmic reticulum stress. Cancers (Basel). 2020;12(3):569. doi:10.3390/cancers12030569 32121537 PMC7139862

[43] Han J, Back SH, Hur J, et al. ER-stress-induced transcriptional regulation increases protein synthesis leading to cell death. Nat Cell Biol. 2013;15(5):481–490. doi:10.1038/ncb2738 23624402 PMC3692270

[44] Shubin AV, Demidyuk IV, Komissarov AA, et al. Cytoplasmic vacuolization in cell death and survival. Oncotarget. 2016;7(34):55863–55889. doi:10.18632/oncotarget.10150 27331412 PMC5342458

[45] Sharma S, Ghufran SM, Ghose S, et al. Cytoplasmic vacuolation with endoplasmic reticulum stress directs sorafenib induced non-apoptotic cell death in hepatic stellate cells. Sci Rep. 2021;11(1):3089. doi:10.1038/s41598-021-82381-3 33542321 PMC7862314

[46] Chen X, Shi C, He M, et al. Endoplasmic reticulum stress: molecular mechanism and therapeutic targets. Signal Transduct Target Ther. 2023;8(1):352. doi:10.1038/s41392-023-01570-w 37709773 PMC10502142

[47] Parzych KR, Klionsky DJ. An overview of autophagy: morphology, mechanism, and regulation. Antioxid Redox Signal. 2014;20(3):460–473. doi:10.1089/ars.2013.5371 23725295 PMC3894687

[48] Yim WW-Y, Mizushima N. Lysosome biology in autophagy. Cell Discov. 2020;6(1):6. doi:10.1038/s41421-020-0141-7 32047650 PMC7010707

[49] Li X, He S, Ma B. Autophagy and autophagy-related proteins in cancer. Mol Cancer. 2020;19(1):12. doi:10.1186/s12943-020-1138-4 31969156 PMC6975070

[50] Sperandio S, Poksay K, De Belle I, et al. Paraptosis: mediation by MAP kinases and inhibition by AIP-1/alix. Cell Death Differ. 2004;11(10):1066–1075. doi:10.1038/sj.cdd.4401465 15195070

[51] Chen X, Chen X, Zhang X, et al. Curcuminoid B63 induces ROS-mediated paraptosis-like cell death by targeting TrxR1 in gastric cells. Redox Biol. 2019;21:101061. doi:10.1016/j.redox.2018.11.019 30590310 PMC6306695

[52] Fontana F, Moretti RM, Raimondi M, et al. δ-Tocotrienol induces apoptosis, involving endoplasmic reticulum stress and autophagy, and paraptosis in prostate cancer cells. Cell Prolif. 2019;52(3):e12576. doi:10.1111/cpr.12576 30719778 PMC6536411

[53] Chang L-C, Chiang S-K, Chen S-E, et al. Exploring paraptosis as a therapeutic approach in cancer treatment. J Biomed Sci. 2024;31(1):101. doi:10.1186/s12929-024-01089-4 39497143 PMC11533606

[54] Ganesan K, Xu C, Wu S, et al. Ononin inhibits tumor bone metastasis and osteoclastogenesis by targeting mitogen-activated protein kinase pathway in breast cancer. Research (Wash D C). 2024;7:0553. 39687715 10.34133/research.0553 PMC11648741

[55] Petrillo S, Chiabrando D, Genova T, et al. Heme accumulation in endothelial cells impairs angiogenesis by triggering paraptosis. Cell Death Differ. 2018;25(3):573–588. doi:10.1038/s41418-017-0001-7 29229999 PMC5864215

[56] Dhani S, Nagiah S, Naidoo DB, et al. Fusaric acid immunotoxicity and MAPK activation in normal peripheral blood mononuclear cells and Thp-1 cells. Sci Rep. 2017;7(1):3051. doi:10.1038/s41598-017-03183-0 28596589 PMC5465181

[57] Sperandio S, Poksay K, de Belle I, et al. Paraptosis: mediation by MAP kinases and inhibition by AIP-1/alix. Cell Death Differ. 2004;11(10):1066–1075. doi:10.1038/sj.cdd.4401465 15195070

[58] Acuña-Pilarte K, Koh MY. The HIF axes in cancer: angiogenesis, metabolism, and immune-modulation. Trends Biochem Sci. 2025;50(8):677–694. doi:10.1016/j.tibs.2025.06.005 40640048 PMC12327054

[59] Liao M, Zhang J, Wang G, et al. Small-Molecule drug discovery in triple negative breast cancer: current situation and future directions. J Med Chem. 2021;64(5):2382–2418. doi:10.1021/acs.jmedchem.0c01180 33650861

[60] Yin L, Duan JJ, Bian XW, et al. Triple-negative breast cancer molecular subtyping and treatment progress. Breast Cancer Res. 2020;22(1):61. doi:10.1186/s13058-020-01296-5 32517735 PMC7285581

[61] Hanker AB, Sudhan DR, Arteaga CL. Overcoming endocrine resistance in breast cancer. Cancer Cell. 2020;37(4):496–513. doi:10.1016/j.ccell.2020.03.009 32289273 PMC7169993

[62] Theodossiou TA, Ali M, Grigalavicius M, et al. Simultaneous defeat of MCF7 and MDA-MB-231 resistances by a hypericin PDT-tamoxifen hybrid therapy. NPJ Breast Cancer. 2019;5:13. 30993194 10.1038/s41523-019-0108-8 PMC6458138

[63] Zare ME, Kansestani AN, Hemmati S, et al. The rate of aerobic glycolysis is a pivotal regulator of tumor progression. J Diabetes Metab Disord. 2021;20(1):523–531. doi:10.1007/s40200-021-00774-7 34178852 PMC8212265

[64] Helal-Neto E, Barcellos-de-Souza P, Morgado-Diaz J, et al. Extracellular matrix derived from high metastatic human breast cancer triggers epithelial-mesenchymal transition in epithelial breast cancer cells through αvβ3 integrin. Int J Mol Sci. 2020;21(8):2995. doi:10.3390/ijms21082995 32340328 PMC7216035

[65] Schito L, Semenza GL. Hypoxia-Inducible factors: master regulators of cancer progression. Trends Cancer. 2016;2(12):758–770. doi:10.1016/j.trecan.2016.10.016 28741521

[66] Rankin EB, Nam JM, Giaccia AJ. Hypoxia: signaling the metastatic cascade. Trends Cancer. 2016;2(6):295–304. doi:10.1016/j.trecan.2016.05.006 28741527 PMC5808868

[67] Semenza GL. Pharmacologic targeting of hypoxia-inducible factors. Annu Rev Pharmacol Toxicol. 2019;59:379–403. doi:10.1146/annurev-pharmtox-010818-021637 30625281

[68] Yang C, Deng X, Tang Y, et al. Natural products reverse cisplatin resistance in the hypoxic tumor microenvironment. Cancer Lett. 2024;598:217116. doi:10.1016/j.canlet.2024.217116. 39002694

[69] Liu Q, Guan C, Liu C, et al. Targeting hypoxia-inducible factor-1alpha: A new strategy for triple-negative breast cancer therapy. Biomed Pharmacother. 2022;156:113861. doi:10.1016/j.biopha.2022.113861. 36228375

[70] Srivastava N, Usmani SS, Subbarayan R, et al. Hypoxia: syndicating triple negative breast cancer against various therapeutic regimens. Front Oncol. 2023;13:1199105. doi:10.3389/fonc.2023.1199105 37492478 PMC10363988

[71] Mendoza EN, Ciriolo MR, Ciccarone F. Hypoxia-Induced reactive oxygen species: their role in cancer resistance and emerging therapies to overcome It. Antioxidants (Basel). 2025;14(1):94. doi:10.3390/antiox14010094 39857427 PMC11762716

